# Molecule-based diagnosis of *Apium graveolens* allergy: is there a need to increase the current allergen panel?

**DOI:** 10.1186/2045-7022-3-S3-O12

**Published:** 2013-07-25

**Authors:** G Gadermaier, E Vejvar, P Briza, K Hoffmann-Sommergruber, W Hemmer, F Ferreira

**Affiliations:** 1 Christian Doppler Laboratory for Allergy Diagnosis and Therapy, University of Salzburg, Salzburg, Austria; 2 Department of Pathophysiology and Allergy Research, Medical University of Vienna, Vienna, Austria; 3 FAZ-Floridsdorf Allergy Center, Vienna, Austria

## Background

Consumption of celery (*Apium graveolens*) has the potential to trigger severe allergic reactions in sensitized patients and thus requires labeling on food products. The aim of the study was to determine the sensitization profile to celery allergens and to identify novel IgE binding proteins in this source.

## Methods

Immunoblots were performed with extracts of celeriac and celery stalks using sera of *A. graveolens* sensitized patients from Austria (n=23). Purified allergens were obtained from natural source (celeriac) or produced as recombinant proteins in *E. coli* and IgE reactivity to rApi g 1, rApi g 2, nApi g 5, and nApi g 6 was tested in ELISA. IgE cross-inhibition assays were performed with purified lipid transfer proteins (LTP). Celeriac extract was separated in 2D gel electrophoresis and IgE reactive spots were subjected to mass spectrometry-based analysis.

## Results

Immunoblots showed distinct IgE reactive bands at 10 and 16 kDa while broad reactivity was observed in the range between 25 and 100 kDa. IgE reactivity to Api g 1, which is solely present in celery tuber and absent in stalks was 60% in *A. graveolens* sensitized patients. Fifty-six percent reacted to Api g 5 while 26-34% of patients were sensitized to Api g 6 (LTP2) and Api g 2 (LTP1) present in celery tuber and stalks, respectively. Interestingly, Api g 6 and Api g 2 sensitization pattern did not correlate and limited IgE cross-reactivity was observed. Eighteen IgE reactive spots from celeriac extract were obtained in 2D gel electrophoresis. Mass analysis identified them as members of the fructose bisphosphate aldolase, malate dehydrogenase, GAPDH, triosephosphate isomerase, chitinase, and manganese superoxide dismutase families.

## Conclusion

*In vitro* diagnosis using purified *A. graveolens* allergens is able to substitute extracts that might give rise to unspecific reactivity. In order to further enhance sensitivity and specificity, newly identified allergens should be produced and included in molecule-based diagnosis of celery allergy.

## Disclosure of interest

None declared.

